# Recurrent Acute Limb Ischemia and Direct Oral Anticoagulant Failure: A Case of Suspected Antiphospholipid Syndrome Triggered by Mechanical Compression

**DOI:** 10.7759/cureus.110899

**Published:** 2026-06-15

**Authors:** Sara Almaradweh, Murali K Manikkavelu, Herbert Oye

**Affiliations:** 1 Medicine, Avalon University School of Medicine, Willemstad, CUW; 2 General Surgery, American University of Antigua, St. John, ATG; 3 Surgery/Endo-Vascular Surgery, Raleigh General Hospital, Beckley, USA; 4 Vascular Surgery, West Virginia Vascular Institute, Beckley, USA

**Keywords:** acute limb ischemia, antiphospholipid syndrome, apixaban failure, arterial thrombosis, ischemia reperfusion injury, recurrent thrombosis, thromboembolectomy

## Abstract

Acute limb ischemia is a vascular emergency that requires prompt diagnosis and treatment to avoid permanent tissue loss and possible amputation. Recurrent arterial thrombosis in a relatively young patient, especially while on anticoagulation, should raise concern for an underlying hypercoagulable state.

We present the case of a 40-year-old woman with type 2 diabetes mellitus, hyperlipidemia, and heavy tobacco use who developed acute right lower extremity ischemia after sitting with her legs crossed for several hours during an episode of severe vomiting and diarrhea. She underwent emergent thromboembolectomy and was discharged on apixaban and aspirin. About two weeks later, she returned with recurrent right lower extremity ischemia despite reported compliance with anticoagulation and was found to have multifocal arterial thrombosis requiring repeat thromboembolectomy and pedal arch angioplasty. Her postoperative leukocytosis was felt to be reactive and related to ischemia-reperfusion injury rather than infection. Because of recurrent arterial thrombosis despite apixaban therapy, further workup for antiphospholipid syndrome was initiated, and she was transitioned to heparin with recommendation for long-term warfarin therapy.

This case highlights that mechanical compression may have contributed to the initial lower extremity ischemic event; however, the subsequent recurrent arterial thromboses, particularly at anatomically distinct sites despite direct oral anticoagulant (DOAC) therapy, were more concerning for an underlying systemic hypercoagulable state such as antiphospholipid syndrome in the setting of additional vascular risk factors.

## Introduction

Acute limb-threatening ischemia (ALTI) represents a vascular emergency that threatens extremity viability, with an incidence of approximately 1.5 cases per 10,000 persons per year [1]. In young and middle-aged populations, ALTI is most frequently associated with embolic events, premature atherosclerosis exacerbated by smoking and diabetes, or hypercoagulable states [2,3]. The standard clinical presentation follows the classic “six Ps”: pain, pallor, pulselessness, paresthesia, paralysis, and poikilothermia [1]. While mechanical thrombectomy has improved limb salvage rates, the standard of care relies on post-procedural anticoagulation, historically vitamin K antagonists (VKAs), and more recently, direct oral anticoagulants (DOACs), to prevent recurrence [2].

A significant gap remains in the literature regarding DOAC efficacy in patients with underlying autoimmune prothrombotic states, such as antiphospholipid syndrome (APS), presenting with arterial events. Recent randomized controlled trials have demonstrated concerning signals regarding worse arterial outcomes with DOACs in patients with thrombotic APS, particularly in those with arterial thrombosis [4,5]. This case highlights recurrent, refractory arterial thrombosis during apixaban therapy in a patient whose clinical presentation raised concern for a possible systemic hypercoagulable state, including suspected antiphospholipid syndrome. By documenting this rare intersection of mechanical provocation, premature atherosclerosis, and recurrent thrombosis while on anticoagulation, this report emphasizes the importance of early hypercoagulable evaluation in young patients presenting with arterial thrombosis.

## Case presentation

A 40-year-old female patient with a history of type 2 diabetes mellitus, hyperlipidemia, and heavy tobacco use presented with acute-onset, excruciating right lower extremity pain. The patient reported that while experiencing acute gastrointestinal distress involving profuse vomiting and diarrhea, she maintained a crossed-leg position for several hours. Shortly thereafter, her right foot exhibited a striking color change from “jaundice yellow” to white, accompanied by profound numbness and burning pain. Initial emergency evaluation confirmed absent Doppler signals in the right foot. She underwent emergent arterial thromboembolectomy for acute limb-threatening ischemia and was discharged on a regimen of apixaban 5 mg twice daily and aspirin 81 mg daily.

**Figure 1:**
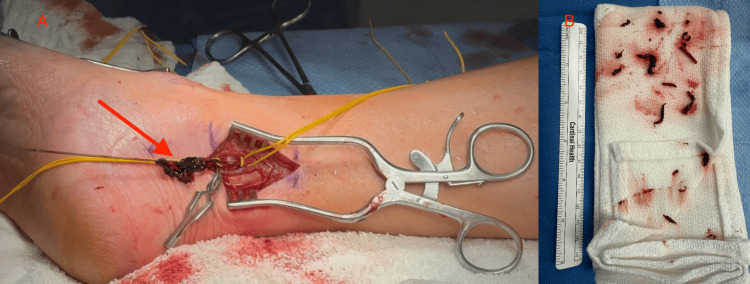
Thrombus removal during repeat intervention for recurrent right lower extremity ischemia (A) Intraoperative image demonstrating thrombus extraction through distal arterial exposure. (B) Gross image of extracted thrombus fragments, shown with a ruler for scale.

Despite adherence to her anticoagulant and antiplatelet therapy, the patient experienced a return of "unrelenting" burning pain and coldness in the right great toe approximately two weeks later. Physical examination revealed a cold right foot with impalpable posterior tibial (PT) and dorsalis pedis (DP) pulses. Computed tomography angiography (CTA) demonstrated extensive atherosclerotic calcification and a lack of continuous contrast enhancement to the ankle, suggestive of multifocal occlusion. Operative documentation further localized the recurrent thrombosis to the right posterior tibial artery at the distal foot/toe level. Given the evidence of recurrent arterial thrombosis while on therapeutic anticoagulation, the patient underwent repeat thromboembolectomy and pedal arch angioplasty (Figure 1).

**Figure 2:**
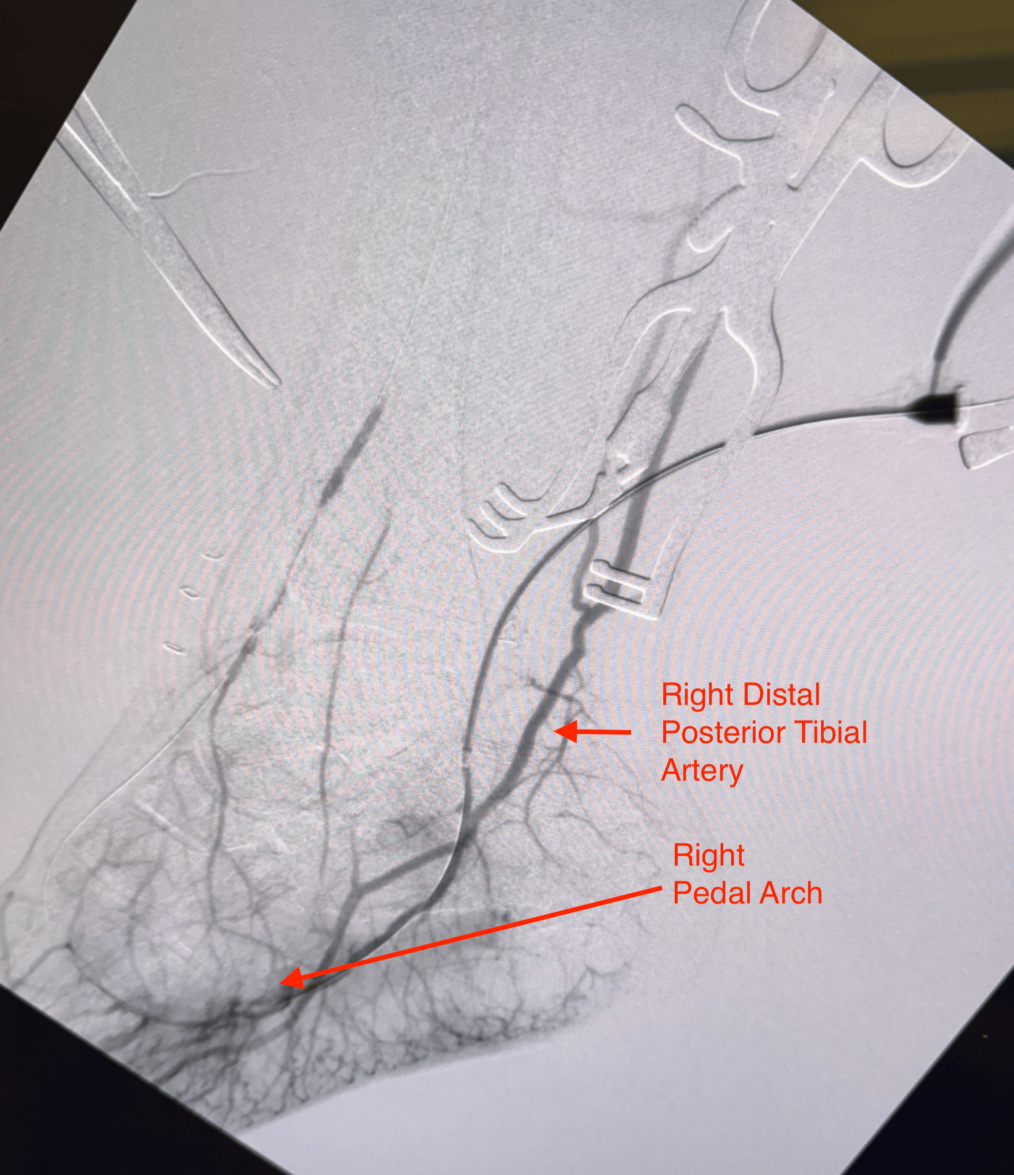
Intraoperative angiographic image demonstrating the patency of the right distal posterior tibial artery and pedal arch following repeat intervention for recurrent right lower extremity ischemia

Intraoperative angiography demonstrated patency of the distal posterior tibial artery and pedal arch following repeat intervention (Figure 2).

Postoperatively, the clinical focus shifted to managing a leukocytosis of 22.2 × 10³/µL. Peripheral blood smear results were not available in the records reviewed. An infectious disease (ID) consultation determined the leukocytosis was reactive to the recent clot burden and ischemia-reperfusion injury rather than infectious, as the count trended downward to 18.3 × 10³/µL without antimicrobial intervention. Hematology/Oncology was consulted for recurrent arterial thrombosis during anticoagulation and recommended thrombophilia testing, including evaluation for antiphospholipid syndrome, lifelong anticoagulation, and transition from apixaban to rivaroxaban. Follow-up thrombophilia and antiphospholipid syndrome results were not included in the available documentation. Given the recurrent arterial thrombosis during anticoagulation in a 40-year-old patient, evaluation for an underlying systemic hypercoagulable state was pursued. Antiphospholipid syndrome was considered in the differential diagnosis, and a thrombophilia profile was ordered, including lupus anticoagulant, anticardiolipin antibodies IgG and IgM, and anti-β2-glycoprotein I antibodies IgG and IgM. Hematology/Oncology recommended long-term anticoagulation, with the final anticoagulation plan pending completion of antiphospholipid syndrome laboratory evaluation.

## Discussion

Recurrent arterial thrombosis on apixaban in suspected antiphospholipid syndrome

The primary challenge in this case is the occurrence of recurrent arterial thrombosis despite documented adherence to apixaban therapy. While DOACs have become standard therapy for venous thromboembolism, accumulating evidence demonstrates their inferiority to warfarin in patients with confirmed APS, particularly those with arterial thrombosis [4,6]. In the present case, antiphospholipid syndrome remained unconfirmed and was considered only as part of the differential diagnosis for a possible systemic hypercoagulable state. A meta-analysis of 4 randomized controlled trials involving 472 patients with thrombotic APS found that DOACs were associated with significantly higher odds of subsequent arterial thrombotic events compared to warfarin (OR 5.43, 95% CI 1.87-15.75), primarily driven by an excess risk of stroke (OR 10.74, 95% CI 2.29-50.38) [4]. Notably, this increased risk was observed across all subgroups, including patients without triple-positive antibody status or prior arterial thrombosis [4].

Current guidelines from multiple societies recommend against the use of DOACs in patients with APS who have either triple-positive antibody status or a history of arterial thrombosis [5-7]. The American Heart Association/American Stroke Association guidelines specifically recommend vitamin K antagonists with a target INR of 2.0-3.0 for patients with arterial thrombosis, with consideration of combination therapy with low-dose aspirin (75-100 mg daily) in select cases [7,8]. Some experts advocate for higher-intensity anticoagulation (INR 3.0-4.0) for arterial events, though randomized trials have not demonstrated superiority of this approach [8,9].

This patient's exclusively arterial presentation with recurrent thrombosis on apixaban is clinically concerning for an underlying prothrombotic state, such as APS, which may overwhelm Factor Xa inhibition through multiple mechanisms, including endothelial activation, complement activation, and enhanced platelet aggregation [9]. Although APS was not confirmed in this case because follow-up laboratory results were unavailable and no relevant obstetric history was documented, the recurrent arterial thrombosis raised concern for a possible systemic hypercoagulable state, with APS remaining part of the differential diagnosis [6,8].

Diagnostic challenges and early clinical suspicion for antiphospholipid syndrome

Diagnosing APS in the acute setting can be challenging because management decisions often must be made before the full diagnostic workup is complete. Although the 2023 ACR/EULAR APS classification criteria provide a standardized framework, they were developed primarily for classification rather than to replace bedside clinical judgment in individual cases [10]. In addition, formal APS classification depends on persistent antiphospholipid antibody positivity over time, which may delay definitive classification even when the clinical presentation is highly suspicious [10,11]. In this patient, recurrent arterial thrombosis despite therapeutic anticoagulation raised concern for an underlying systemic hypercoagulable state, making it reasonable to include APS in the differential diagnosis while awaiting confirmatory testing.

Interpretation of the laboratory evaluation may also be complicated in patients who are already receiving anticoagulation, particularly when lupus anticoagulant testing and anticoagulation monitoring are performed during active treatment [11,12]. For that reason, suspected APS should not be approached as a purely laboratory diagnosis, especially when the presentation involves recurrent arterial thrombosis in a relatively young patient. This case highlights the importance of considering systemic hypercoagulable states, including APS, when the clinical pattern is disproportionate to the patient’s age, and thrombosis occurs despite apparently appropriate anticoagulant therapy [10-12].

Leukocytosis as a marker of ischemia-reperfusion injury

The marked leukocytosis (22.2 × 10³/µL) initially raised concern for infection, a critical differential given the potential for limb-threatening complications. However, the ID evaluation correctly identified this as reactive leukocytosis secondary to tissue ischemia and reperfusion injury rather than an infectious process. This distinction is essential for appropriate antibiotic stewardship.

An ischemia-reperfusion injury triggers a profound inflammatory cascade characterized by enhanced leukocyte-endothelial cell adhesion, leukocyte trafficking into tissues, and release of inflammatory mediators [13,14]. During the reperfusion phase, activated endothelial cells produce increased oxygen radicals while generating less nitric oxide, creating an imbalance that promotes the biosynthesis of adhesion molecules and release of inflammatory mediators such as platelet-activating factor and tumor necrosis factor [13,15]. This results in leukocyte plugging in capillaries and enhanced leukocyte trafficking in postcapillary venules [13,16].

The release of damage-associated molecular patterns (DAMPs) from ischemic myocytes triggers neutrophil mobilization and release from bone marrow, manifesting as peripheral leukocytosis [13,17]. This reactive leukocytosis can mimic sepsis but represents a sterile inflammatory response to tissue injury [13]. The downward trend in white blood cell count without antimicrobial intervention supports this interpretation and demonstrates appropriate clinical judgment in avoiding unnecessary antibiotic exposure.

Mechanical provocation in the setting of atherosclerotic disease

The mechanical trigger of prolonged leg crossing during an episode of vomiting represents a plausible "second hit" to this patient's underlying atherosclerotic disease. The CTA findings of extensive atherosclerotic calcification suggest chronic vascular disease, likely exacerbated by her significant risk factors, including heavy tobacco use, diabetes mellitus, and hyperlipidemia [2,18]. Young patients with peripheral artery disease demonstrate distinct risk factor profiles, with higher rates of active smoking, lower HDL cholesterol, and elevated lipoprotein(a) compared to older patients [18].

The combination of mechanical compression, underlying stenotic disease, and potential hypercoagulability may have created a "perfect storm" for acute thrombosis. This mechanism serves as a practical clinical reminder that mechanical factors can precipitate terminal perfusion failure in patients with existing vascular disease, even in the absence of complete arterial occlusion [1,2]. In this case, the crossed-leg position may have reduced arterial inflow through already compromised distal vessels and promoted thrombosis in a low-flow state.

Risk stratification and long-term management

This case underscores several important principles for managing young patients with acute limb ischemia. First, the presence of recurrent arterial thrombosis despite therapeutic anticoagulation should prompt immediate evaluation for systemic hypercoagulable states, particularly APS [8,19,20]. The 2023 ACR/EULAR APS classification criteria require at least one positive antiphospholipid antibody test within 3 years of an aPL-associated clinical criterion, with weighted scoring across clinical and laboratory domains [10]. While classification criteria are designed for research cohorts, diagnostic assessment in clinical practice should be broader to optimize patient management [11].

Second, young patients (≤50 years) with de novo arterial thrombosis or embolism demonstrate worse short-term limb-related outcomes compared to older patients, with higher rates of major adverse limb events and reintervention [3]. This paradoxical finding may relate to the absence of protective collateral circulation that develops in chronic atherosclerotic disease, as well as different underlying etiologies, including hypercoagulable states and non-atherosclerotic arteriopathies [3].

Third, aggressive cardiovascular risk factor modification is essential. This patient's heavy tobacco use represents the most modifiable risk factor, with smoking cessation being paramount [2,18]. Optimal management of diabetes and hyperlipidemia, including high-intensity statin therapy, is recommended by current guidelines [2]. Long-term surveillance for recurrent events and monitoring of anticoagulation intensity will be critical, particularly given the challenges of warfarin monitoring in patients with lupus anticoagulant positivity [12].

## Conclusions

This case illustrates that rapid, recurrent arterial thrombosis during direct oral anticoagulant therapy should prompt evaluation for a broad differential, including systemic hypercoagulable states, vasculitis or other inflammatory arteriopathies, embolic sources, malignancy, and progression of atherosclerotic disease, particularly in relatively young patients with arterial events. In patients with confirmed thrombotic antiphospholipid syndrome, evidence from randomized controlled trials has shown increased arterial thrombotic events with direct oral anticoagulants compared with warfarin, supporting current guideline recommendations against direct oral anticoagulant use in that population. In the present case, antiphospholipid syndrome remained suspected but unconfirmed; therefore, recurrent thrombosis should be interpreted as a trigger for broader diagnostic evaluation rather than definitive antiphospholipid syndrome-related direct oral anticoagulant failure.
